# Emerging Roles of Mitogen-Activated Protein Kinase Signaling Pathways in the Regulation of Fruit Ripening and Postharvest Quality

**DOI:** 10.3390/ijms25052831

**Published:** 2024-02-29

**Authors:** Juan Jin, Wei Wang, Dingyu Fan, Qing Hao, Wensuo Jia

**Affiliations:** 1 Institute of Horticulture Crops, Xinjiang Academy of Agricultural Sciences, Urumqi 830000, China; jinjuan316@126.com (J.J.); fandingyu2413@163.com (D.F.); haoqingxj@sohu.com (Q.H.); 2 College of Horticulture, China Agricultural University, Beijing 100193, China; w@cau.edu.cn

**Keywords:** MAPK, signal transduction, fruit ripening, postharvest quality

## Abstract

Fleshy fruit ripening is a unique biological process that involves dramatic changes in a diverse array of cellular metabolisms. The regulation of these metabolisms is essentially mediated by cellular signal transduction of internal (e.g., hormones) and external cues (i.e., environmental stimuli). Mitogen-activated protein kinase (MAPK) signaling pathways play crucial roles in a diverse array of biological processes, such as plant growth, development and biotic/abiotic responses. Accumulating evidence suggests that MAPK signaling pathways are also implicated in fruit ripening and quality formation. However, while MAPK signaling has been extensively reviewed in *Arabidopsis* and some crop plants, the comprehensive picture of how MAPK signaling regulates fruit ripening and quality formation remains unclear. In this review, we summarize and discuss research in this area. We first summarize recent studies on the expression patterns of related kinase members in relation to fruit development and ripening and then summarize and discuss the crucial evidence of the involvement of MAPK signaling in fruit ripening and quality formation. Finally, we propose several perspectives, highlighting the research matters and questions that should be afforded particular attention in future studies.

## 1. Introduction

Fruit ripening and quality formation are two tightly coupled biological events. Fruit ripening is a complex process that involves dramatic changes in cellular metabolisms, thereby laying the basis for fruit quality formation, such as sugar, acid, color, texture, flavor and shelf life [1,2]. In recent years, substantial efforts have been devoted to understanding the regulatory mechanisms behind fruit ripening and quality formation, among which hormonal regulation has been a particular focus. According to the pattern of physiological changes, fleshy fruits can be categorized into two major groups, i.e., climacteric (CL) and non-climacteric fruits (NC). CL fruits are characterized by a burst of respiration and ethylene (abbreviated as ET hereafter) production during fruit ripening, whereas accumulating evidence suggests that NC fruit ripening is regulated by multiple hormones, with abscisic acid (ABA) as a major regulator [3,4,5,6].

The hormonal regulation of fruit ripening is essentially a cellular signal transduction process [6,7,8] that links the perception of hormones with the regulation of the enzymes that control fruit ripening-associated metabolisms. Enzymatic regulation can be achieved transcriptionally and post-transcriptionally. Transcriptional regulation is largely determined by transcription factors (TFs), whereas post-transcriptional regulation is achieved by the group modification of the topological structure, which may result in changes in many protein behaviors, such as activity, stability and cellular localization [3,9,10]. Protein kinase/phosphatase-catalyzed reversible phosphorylation has been established to be the major mechanism of protein modification and therefore plays a pivotal role in cellular signal transduction [11,12]. In recent decades, extensive efforts have been made to explore the transcriptional regulation mechanisms of cellular metabolisms. Although a large number of TFs together with their target genes have been identified to be involved in specific metabolic systems, the profound signaling mechanisms upstream of TFs remain largely elusive [12]. In recent years, explorations into the mechanisms of signal transduction in fruit ripening have attracted the attention of plant scientists. A summary and discussion of the updated knowledge in this area will contribute to further elucidating the regulatory mechanisms of fruit ripening and quality formation.

Mitogen-activated protein kinase (MAPK) cascades are highly conserved signaling mechanisms in eukaryotes [13,14]. They are encoded by a large family of serine/threonine protein kinases [15]. Typically, MAPKs are activated by upstream MAPK kinases (MAPKKs or MEKs), which are in turn phosphorylated and activated by MAPK kinase kinases (MAPKKKs or MEKKs). Consequently, it has been generally thought that the three interlinked protein kinases, MAPKKK-MAPKK-MAPK, constitute a basic module in a specific set of MAPK pathways [14,16,17,18]. For the convenience of discussion, this component of the MAPKKK-MAPKK-MAPK signaling module is hereafter referred to as MAPs kinases (where ‘s’ denotes any number of ‘K’).

While a large number of studies suggest that MAPK signaling is involved in a diverse array of biological processes, particular interest has been paid to the role of MAPK signaling in hormone signaling and plant immunity [18,19,20,21,22,23]. It has been suggested that MAPK signaling is involved in the signaling of all hormones, such as ET, ABA, SA, JA and IAA [20,23]. Intriguingly, MAPK signaling was reported to be related not only to hormonal signal transduction but also to hormonal biosynthesis. In *Arabidopsis thaliana*, for example, MPK3 and MPK6 were shown to be able to phosphorylate and modulate the stability of EIN3, a key transcriptional regulator of ET-induced transcriptional machinery [24]; meanwhile, they were also shown to be able to phosphorylate and modulate the stability of two members of ACC synthases (ACS2 and ACS6), thereby promoting ET production [23,25,26]. These studies demonstrated that MAPs kinases play pivotal roles in both ET signal transduction and biosynthesis. ET is known to be a key regulator of CL fruit ripening. Accordingly, MAPK signaling is also likely to be involved in fruit ripening, supposing that ET signal transduction shares a common mechanism in different plants/organs. Consistent with this speculation, accumulating evidence suggests that MAPK signaling does indeed play important roles in fruit ripening and quality formation [27,28,29,30,31]. In recent decades, both fruit ripening and MAPK signaling have been reviewed extensively [18,19,20,21,22,23]; however, the role of MAPK signaling in fruit ripening and quality formation has never been reviewed. This review aims to bring together recent advances in our knowledge of MAPK signaling in fruit ripening and quality formation. 

## 2. Recent Studies on Patterns of Gene Expression of MAPs Kinases in Relation to Fruit Development and Ripening

A number of studies have been conducted on the patterns of gene expression of MAPs kinases in fruit development and ripening in different plant species (32–40). Analysis of the *Arabidopsis* genome revealed the presence of 20 *MAPK*s, 10 *MAPKK*s and around 80 *MAPKKK*s [32]. Bioinformatical studies have suggested that MAPs kinase membership composition patterns are basically similar among different plant species. Unfortunately, no comprehensive profile of the gene expression of MAPs kinase or information on fruit development and ripening in several representative fruit species is available.

In contrast to *Arabidopsis thaliana*, in which a transgenic system has been well established, most Fleshy-Fruited Plants (FFPs) except for tomatoes and strawberries lack a transgenic system, and this has been a major limitation in identifying the MAPs kinase implicated in the regulation of fruit development and ripening. Although studies on the patterns of gene expression have not able to conclusively demonstrate the essential roles of specific MAPs kinases in fruit development and ripening, a comprehensive understanding of the spatiotemporal profile of MAPs kinase gene expression would be able to provide important information for identifying the potential genes involved in fruit development and ripening. Accordingly, in the current review, we summarize and update knowledge on bioinformatics and gene expression analysis in both CL and NC plants in order to understand the comprehensive picture of MAPK signaling in relation to fruit ripening and quality formation.

The tomato (*Solanum lycopersicum*) is known as a model CL fruit. An early study identified 16 *MAPK* members in tomatoes, denoted as *SlMAPK1-16*, most of which could be detected in the leaves, stems, flowers and fruit [33]. Although nearly all members could be detected in the fruit and leaves, no members were preferentially expressed in the fruit, whereas a few members (e.g., *SlMPK3/6/9*) were found to be preferentially expressed in the leaves. A subsequent study by Wu et al. (2014) [34] identified five *SlMAPKK*s and 89 *SlMAPKKK*s in the tomato genome. Most of these genes were constitutively expressed in the leaves, stems, flowers and fruit. Twelve *SlMAPKKK*s showed higher expression levels in the roots than in the other organs, whereas only three *SlMAPKKK*s (*SlMAPKKK33*, *SlMAPKKK34* and *SlMAPKKK35*) were expressed at a high abundance in fruits.

Apples and bananas are not only economically important horticultural crops but also typical CL fruits. An early study by Zhang et al. (2013) [35] identified 26 putative *MAPK* genes and nine *MAPKK* genes in the apple genome (*Malus domestica*). Among the 26 *MAPK* genes, 8 genes were expressed in all tissues while 17 showed different tissue-specific expression profiles. Moreover, while both the leaves and roots showed the presence of genes with tissue-specific expression (for example, *MdMPK13-2* and *MdMPK20-2* were exclusively expressed in the roots, and *MdMPK1-1* was preferentially expressed in the leaves) no genes were found with a pattern of exclusive or preferential expression in the fruits. ET is well known as the central signal that controls CL fruit ripening [6,36]. Asif et al. (2014) [37] studied the effect of ET on the expression of *MAPK*s in bananas (*Musa acuminate*), a typical CL fruit. Among the 25 *MaMAPK*s identified in the banana genome, a set of *MaMAPK*s were highly expressed during fruit ripening and senescence, and 8 *MaMAPK*s were reduced by ET treatment, suggesting that many *MaMAPK*s might be involved in the regulation of banana fruit ripening. Subsequently, a study by Wang et al. (2017) [38] identified the presence of a total of 10 *MAPKKs* (*MaMKKs*) and 77 *MAPKKKs* in the banana genome. Expression analysis indicated that the transcript levels of most of these genes were much higher in the early stage (i.e., 0 and 20 DAF) than in the late stage (i.e., 80 DAF and postharvest), whereas a few genes showed significantly higher expressional levels in the late stage (80 DAF and postharvest) than in the early stage (0 and 20 DAF), e.g., *MaMAPKKK38*, *MaMKK4* and *MaMKK1*, suggesting that some genes might play a pivotal role in the regulation of banana fruit development and ripening.

The strawberry is emerging as a model NC fruit. A study by Shulaev et al. [39] identified a total of 12 *MAPK*, seven *MAPKK*, 73 *MAPKKK* and one *MAP4K* putative MAPK cascade proteins in the *Fragaria vesca* genome. The expression patterns of *FvMAPK*s and *FvMAPKK*s were subsequently analyzed by Zhou et al. (2017) [40]. Noteworthily, while the presence of genes that were exclusively or preferentially expressed in leaves was identified, such as *FvMAPK10* and *FvMAPKK6/7*, no genes were exclusively or preferentially expressed in fruit, and some genes (such as *FvMAPKK1/3/6/7*) were not expressed in fruit. Among the twelve *FvMAPK*s, two genes (*FvMAPK2* and *FvMAPK12*) showed much lower expression than the others in all the developmental stages. The expression of six genes (such as *FvMAPK5*, *FvMAPK6*, *FvMAPK7* and *FvMAPK8*) first declined in the early stage and then increased in the later stages of fruit development; meanwhile, three genes (*FvMAPK3*, *FvMAPK4* and *FvMAPK11*) increased and another three genes (*FvMAPK1*, *FvMAPK9* and *FvMAPK10*) declined throughout the whole developmental process. Strikingly, in the *FvMAPKK*s subfamily, only *FvMAPKK2*, *FvMAPKK4* and *FvMAPKK5* were expressed in fruits at a detectable level and gradually increased through fruit development. In terms of the *FvMAPKKK* subfamily, the 73 members were divided into three major groups according to their expression patterns. One group showed high expression in the early stage, which gradually decreased throughout fruit development and ripening, another group showed the opposite pattern, whereas the third group showed relatively higher expression in the middle stage compared with both the early and later stages. ABA, JA and sucrose have been established to be key regulators of strawberry fruit ripening [41]. It was reported that most of the *MAPK* members can be induced by ABA, JA and sucrose [42,43]. 

Besides the strawberry, several other NC fruits, such as grapevine (*Vitis vinifera*), kiwi fruit (*Actinidia deliciosa*), and jujube (*Ziziphus jujuba Mill.*), were also studied to determine their MAPs kinase patterns of gene expression. In the jujube genome, nine typical *MAPK*s and five *MAPKK*s were identified, and most of these genes were expressed in all the tested organs/tissues, e.g., roots, bearing shoots, secondary shoots, leaves, flower buds, flowers and fruit. Similar to the strawberry, no *MAPK* members were exclusively or preferentially expressed in jujube fruit. In contrast to *MAPK*s, some *MKK*s showed a pattern of tissue/organ-specific expression. For example, *ZjMKK5* was specifically expressed in flower buds, whereas *ZjMKK2* was largely expressed in fruits. A subsequent study identified a total of 56 *ZjMAPKKK*s, but unfortunately, their expression profile in fruit development was not reported [44]. In grapevines, Wang et al. (2014) [45] identified 45 *VviMAPKKK* genes in the grapevine genome. All *VviMAPKKK* members were expressed in at least one developmental stage of grape organs, such as flowers, berries, buds, leaves, rachis, roots, seeds, seedlings, stems and tendrils. Generally, the genes were much higher in young tissues and organs than in ripening or senescing ones, suggesting that these *VviMAPKKK*s are mostly related to signal transduction during development in metabolically active tissues. These results provide important information for further elucidating the signaling mechanism of *VviMAPKKK*s in grapevine developmental biology. 

Collectively, studies on the gene expression of MAPs kinases have provided important clues for their involvement in fruit development, ripening and quality formation. In terms of subfamily composition, the different plants reported showed a similar pattern to *Arabidopsis thaliana*, i.e., around 20 (10~30) *MAPK*s, 10 *MAPKK*s (5~10) [32] and 80 (40–90) *MAPKKK*s [32]. For *MAPK*s and *MAPKK*s, nearly all the members were expressed in fruit, but no members were exclusively or preferentially expressed in fruit. In contrast to *MAPK*s and *MAPKK*s, a few *MAPKKK*s were reported to be preferentially expressed in fruit, although the expression of *MAPKKKs* was generally much higher in young tissues and organs than in ripening or senescing ones. This may be related to the pattern of membership composition of the *MAPKKK/MAPKK/MAPK* families, i.e., in comparison with *MAPK*s and *MAPKK*s, the number of *MAPKKK* members is much larger (80 members in *Arabidopsis*). Thus, it is logical that some individual members of *MAPKKK*s are specifically involved in the regulation of some specific biological processes. By contrast, the number of *MAPKK*s is significantly lower (only 10 in *Arabidopsis*), and as such, it is not reasonable for individual *MAPKK*s to be involved in specific biological processes. Accordingly, more attention should be paid to the role of *MAPKKK*s in fruit development and ripening. Noteworthily, most of these studies have focused on patterns of gene expression in different organs [32,33,35,39,40]. It is the changing pattern, along with fruit development and ripening, rather than the absolute level in a specific stage of the MAPK signaling members, that may be more valuable for exploring the mechanisms of fruit development and ripening. Unfortunately, except for a report on strawberries by Zhou et al. (2017) [40], as described above, the information about changing patterns in the gene expression of *MPK* kinases throughout the entire developmental process remains largely unclear.

## 3. Roles of MAPK Signaling in Fruit Development and Ripening as Revealed by Loss-of-Function and Gain-of-Function Studies

Loss of function and gain of function are basic strategies for the functional identification of genes. They can be realized using several technologies. As far as fruit studies are concerned, the most commonly used technologies are gene knockdown/out and overexpression. Loss-of-function and gain-of-function studies strongly suggest that a number of MAPK signaling components, modules and pathways play crucial roles in the regulation of a diverse assay of biological processes in fruit ripening and quality formation, as summarized and discussed below.

### 3.1. MAPK Signaling Mediates Ripening-Associated Metabolisms

An early study of tomatoes showed that MAPK pathway inhibitor U0126 treatment resulted in a reduction in fruit ET, proline production, fruit firmness and cold tolerance. Moreover, in response to cold and ethephon treatment, the ET content, the activity of ACS and ACO and the expression of *LeACS2*, *LeACO1* and *LeMAPK4* increased, indicating that MAPK signaling was involved in the regulation of ET production and a variety of ripening-associated metabolisms [46]. More recently, a study by Shu et al. (2023) [47] reported that cold treatment promoted *SlMAPK3* expression in tomato fruit in an ET-dependent manner; meanwhile, the overexpression of *SlMAPK3* promoted ET production and the expression of ET-, cold- and heat-responsive genes in tomato fruits [47]. In strawberries, a study by Mao et al. (2021) [27] reported that low temperatures inhibited anthocyanin metabolism. The results of their study showed that FvMPK3 could phosphorylate and enhance the degradation of FvMYB10, a key TF controlling anthocyanin accumulation. As the activity of FvMPK3 could be induced by low temperature, the authors proposed that the low temperature-induced inhibition of anthocyanin accumulation resulted from the activation of FvMPK3. Noteworthily, many MAPKs have been commonly reported to be activated transcriptionally or post-transcriptionally in different plant species [48,49,50,51,52,53]. In particular, FvMPK6, a MAPK with a similar structure and function to FvMPK3, was reported to be sensitively activated by high temperatures in strawberries. This has raised the question of whether high temperature-induced anthocyanin accumulation might be related to FvMPK3. To clarify this, the pattern of FvMPK3 responses to high-temperature stress needs to be investigated. In *Arabidopsis*, AtMPK3 was reported to be activated by ABA [54,55]. Given that ABA is a key regulator of strawberry fruit ripening, it is important to elucidate whether ABA-induced anthocyanin accumulation is correlated to FvMPK3 in strawberry fruit ripening.

In apples (*Malus domestica*), a study by Sun et al. (2022) [56] reported that low-nitrogen conditions could induce anthocyanin synthesis in apple callus and promote the expression of mitogen-activated protein kinase 9 (*MdMKK9*). CRISPR/Cas9 mutation of *MdMKK9* compromised low-nitrogen-induced anthocyanin biosynthesis. Conversely, the overexpression of *MdMKK9* induced anthocyanin synthesis and a set of anthocyanin metabolism-associated genes, suggesting that *MdMKK9* was implicated in the modification of color metabolism via nitrogen availability. *HY5* plays crucial roles in light-induced anthocyanin accumulation. A more recent study reported that the mitogen-activated protein kinase MdMPK6 could interact with and phosphorylate MdHY5 in response to light signaling. Light-activated MdMPK6 phosphorylated MdHY5, leading to the anthocyanin accumulation of related genes in apple fruit [57]. In the banana (*Musa acuminata*), Wu et al. (2019) [29] identified a basic leucine zipper (bZIP) TF, *MabZIP93*, and showed that *MabZIP93* acted to control the expression of a set of genes implicated in cell wall metabolisms, such as *MaPL2*, *MaPE1*, *MaXTH23* and *MaXGT1*. Transient overexpression of *MabZIP93* in banana fruit promoted the expression of *MaPL2*, *MaPE1*, *MaXTH23* and *MaXGT1*. The mitogen-activated protein kinase MaMPK2 could interact with and phosphorylate MabZIP93. These observations suggest that the MaMPK2-MabZIP93 signaling module functions in the regulation of cell wall metabolism during banana fruit ripening [29].

### 3.2. MAPK Signaling Targets Ripening-Associated TFs

In the banana (*Musa acuminata*), through phosphoproteomic analysis during fruit ripening, Wu et al. (2022) [58] identified *MabZIP21*, a basic leucine zipper TF 21, which could be phosphorylated by MaMPK6-3. Transient overexpression of the phosphomimetic form of *MabZIP21* accelerated banana fruit ripening. These results suggested that the MaMPK6-3-MabZIP21 signaling module played a role in the regulation of banana fruit ripening [58]. Subsequently, the authors demonstrated that MaMKK1 was implicated in the regulation of banana fruit ripening. Transient overexpression or silencing of *MaKK1* in fruit, respectively, accelerated and delayed banana fruit ripening. MaMKK1 could interact with and phosphorylate MaMPK6-3 and MaMPK11, thereby activating the MPK kinases, and MaMPK11-4 could in turn phosphorylate MabZIP21 to regulate its transcription activity. As such, the authors identified the signaling pathway of the MaMPK6-3/11-4-MabZIP21 module that was implicated in the regulation of banana fruit ripening [28].

In the apple (*Malus domestica*), a study by Wei et al. (2023) [59] reported that the activity of MdMAPK3 was activated by ET. MdMAPK3 could interact with and phosphorylate MdNAC72, a NAM-ATAF1/2-CUC2 72 TF, which acted to repress the expression of the cell-wall-degradation-related gene POLYGALACTURONASE1 (*MdPG1*). Moreover, the protein stability of MdNAC72 was modulated by MdMAPK3-targeted phosphorylation in response to ET signaling via an E3 ubiquitin ligase pathway. Transient overexpression of *MdMAPK3* promoted fruit ripening and, conversely, transiently silenced *MdMAPK3* in fruit showed the phenotype opposite to *MdMAPK3*-OE fruit during storage. These results demonstrated that the MdMAPK3-MdNAC72 signaling module plays an important role in apple fruit ripening [59]. A study by Yang et al. (2022) [31] showed that the protein level of the TF MaMYB4 decreased along with banana fruit ripening, which was coupled with ET production and a decline in firmness. MaMYB4 could bind to the promoters of a series of ripening-associated genes, including ET biosynthetic and cell-wall-modifying genes. In addition, the authors found that the protein levels of the two RING finger E3 ligases MaBRG2/3 increased with fruit ripening; moreover, they could interact with and ubiquitinate MaMYB4 to promote its degradation. Collectively, these results suggest that MaMYB4 negatively modulated banana fruit ripening [31]. A further study suggested that transient overexpression of *MaMPK14* and *MaMYB4* delayed fruit ripening. *MaMYB4* represses the expression of the genes involved in ET biosynthesis and fruit softening, such as *MaACS1*, *MaXTH5*, *MaPG3* and *MaEXPA15*. Moreover, MaMPK14 was shown to be able to phosphorylate MaMYB4 at Ser160 to reduce the interaction with MaMPK14 [60].

Yang et al. (2021) [30] reported that the expression of two *MdMPK4* genes was induced by light. MdMPK4 could interact with MdMYB1, and the overexpression of *MdMPK4* and *MdMYB1* promoted anthocyanin accumulation in apple (*Malus domestica*) fruit peels, suggesting that the MdMPK4-MdMYB1 signaling module is implicated in light-induced anthocyanin accumulation [30].

In summary, it has been increasingly suggested that the loss of function or gain of function of some members of MAPs kinases may be able to alter the progress of fruit ripening. However, it should be noted that fruit ripening is orchestrated by dramatic changes in diverse cellular metabolisms. Most of the studies on MAPK signaling in relation to fruit ripening have focused on the effects of loss of function and gain of function in MAPs kinase on the accumulation of anthocyanin. To comprehensively demonstrate the role of MAPK signaling in fruit ripening, studies on other related metabolisms, such as sugar, acid, texture, flavor, etc., are required. Also, owing to the lack of stable transgenic techniques for most FFPs, many loss-of-function and gain-of-function studies of fruit have used unreliable transient expression systems to draw associated conclusions. Regardless, current studies have provided strong evidence for the involvement of MAPK signaling in the regulation of fruit ripening.

## 4. Roles of MAPK Signaling in the Regulation of Fruit Disease Resistance as Demonstrated by Loss-of-Function and Gain-of-Function Studies

Fruit postharvest quality is determined by changing patterns of cellular metabolisms during fruit ripening, such as sugar, acid, color, flavor, texture, etc. Besides these common quality parameters, fruit storage quality is especially important because of the huge economic loss caused by fruit rot worldwide. Although MAPK signaling is involved in the regulation of a diverse array of biological processes, a major role of MAPK signaling is in mediating plant defense responses [19,22,61,62]. Accordingly, it is likely that MAPK signaling plays a crucial role in the formation of fruit storage quality. In support of this speculation, a number of studies have provided evidence for the important role of MAPK signaling in fruit disease resistance. For example, a pharmacological study showed that the application of acibenzolar-S-methyl (ASM), a functional analog of salicylic acid, and the mitogen-activated protein kinase (MAPK) inhibitor PD98059 to apple fruit provided resistance to infection by *Penicillium expansum*. ASM treatment inhibited lesion growth and the activities of a series of ROS-scavenging enzymes, resulting in the elevation of H_2_O_2_ content. Meanwhile, ASM treatment promoted the expression of *MdMAPK4*, *MdMAPK2* and *MdMAPKK1*, whereas it suppressed the expression of *MdMAPK3*. PD98059 +ASM treatment increased CAT activity. These findings indicate that MAPK cascades are implicated in ASM-induced apple fruit resistance to *Penicillium expansum* [63].

FERONIA (FER) is a receptor-like kinase that plays important roles in diverse biological processes. It has been demonstrated that FER regulates fruit ripening [64,65]. In the tomato (*Solanum lycopersicum*), a recent study by Ji et al. (2023) [66] showed that SlFERL, a receptor-like-kinase, interacted with the secreted virulence protein BcPG1 from *Botrytis cinerea*. SlFERL acts to trigger downstream signaling by phosphorylating SlMAP3K18 at Thr45, Ser49, Ser76 and Ser135. SlMAP2K2 and SlMAP2K4 were shown to be related to the immune response of tomatoes to *Botrytis cinerea*. Furthermore, the phosphorylation of SlMAP2K2/SlMAP2K4 by SlMAP3K18 modulated protein stability and kinase activity. Thus, this study identified a signaling cascade: BcPG1-SlFERL-SlMAP3K18-SlMAP2K2/SlMAP2K4-immuno response to *Botrytis cinerea* invasion. Li et al. (2021) [67] reported that the application of exogenous β-aminobutyric acid (BABA) could induce TGA1-related systemic acquired resistance (SAR), thereby alleviating Rhizopus rot in postharvest peach fruit. Upon treatment with BABA, the expressions of a set of redox-regulated genes were promoted, and the activity of *PpTGA1*, a key redox-controlled factor of SAR essential for the activation of priming resistance in postharvest peach fruit [68], was elevated. PpMAPKK5 was shown to interact with and regulate the DNA-binding activity of PpTGA1 for the activation of salicylic acid (SA)-responsive PR genes. The heterologous expression of *PpMAPKK5* in *Arabidopsis* conferred resistance against the fungus *Rhizopus stolonifer*. These observations suggest that the PpMAPKK5-PpTGA1 module has a disease-resistance function in postharvest peach fruit [67]. In plants, MAPK cascades have a critical role in the regulation of plant immunity and disease resistance. Although the function of MAPK cascades in immunity regulation is partially conserved between different species, the mechanisms vary in different host and pathogen combinations. To date, the MAPK cascade function of woody plants in the regulation of disease resistance has seldom been reported. A study by Wang et al. (2022) [69] showed that *Botryosphaeria dothidea* infection induced *MdMAPKKK1* expression in apple fruit. The overexpression of *MdMAPKKK1* induced pathogen-independent cell death, thereby increasing fruit resistance to *B. dothidea*, and conversely, *MdMAPKKK1* silencing reduced fruit resistance to *B. dothidea*. MdMAPKKK1 could interact with and be phosphorylated by MdBSK1, a brassinosteroid-signaling kinase protein. *MdBSK1* silencing also reduced fruit resistance to *B. dothidea*, implying that the MdBSK1- MdMAPKKK1 signaling module may play an important role in apple fruit resistance to *B. dothidea* [69].

In summary, it has been established that MAPK signaling plays crucial roles in the defense responses of *Arabidopsis* and some crop plants [19,22,61,62]. Similarly, it has been increasingly reported that MAPK signaling may also play an important role in FFP resistance to biotic stresses as discussed above. Fruit shelf life constitutes an essential part of fruit quality. While reports on the role of MAPK signaling in FFP disease resistance are accumulating, studies on the role of MAPK signaling in fruit shelf life are still very limited. Furthermore, fruit shelf life is not only determined by fruit disease resistance but also by the ripening progress. Given that MAPK signaling may play an important role in FFP disease resistance and fruit ripening, to decipher the mechanism of MAPK signaling in relation to fruit shelf life, a profound analysis of the distinct roles of MAPK signaling in fruit disease resistance and ripening is required.

**Figure 1:**
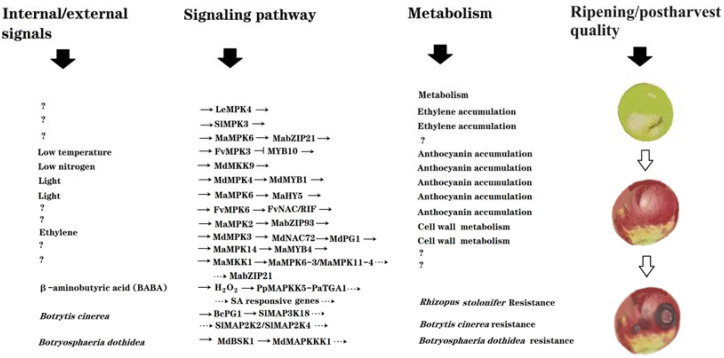
Diagram showing the updated signaling components and pathways of MAPs kinases identified as being implicated in the regulation of fruit ripening and postharvest quality. Fruit ripening and postharvest quality are determined by internal and external signals or, alternatively, by hormonal and environmental signals. Fruit ripening and quality are essentially determined by complicated cellular metabolisms, such as color, sugar, acid, flavor, texture, etc. In addition, anti-pathogenic activity is also an important part of fruit quality, as it determines the postharvest shelf life of fruits. MAPK signaling mediates the internal/external signal-triggered regulation of fruit ripening and quality-associated metabolisms. ‘?’ denotes currently unidentified components/biological events; ‘→’ denotes activation; and a horizontal ‘⊥’ denotes inhibition. Plant species are as follows: ‘Sl’ for tomato ‘*Solanum lycopersicum*’; ‘Le’ for tomato ‘*Lycopersicon esculentum*’; ‘Ma’ for banana ‘*Musa acuminata*’; ‘Fv’ for strawberry ‘*Fragaria vesca*’; ‘Md’ for apple ‘*Malus domestica*’.

As summarized in Figure 1, a number of MAPs kinases have been identified to be involved in fruit ripening and quality formation. Moreover, some specific signaling modules and pathways of MAPs kinases have been elucidated. As most of them were identified via loss-of-function and gain-of-function studies, it can be concluded that MAPK signaling plays essential roles in the regulation of fruit ripening and quality formation. Nevertheless, current information in this research area is still very limited, and the profound mechanisms remain largely unknown. As described in Section 5, we have proposed several points and questions that should be given special consideration in future studies.

## 5. Conclusions and Perspectives

### 5.1. Systemic Study on MAPs Kinase Gene Expression Patterns in Relation to Fruit Ripening

In recent decades, many reports have been published on the gene expression of MAPs kinases in different FFP species (Fleshy-Fruited Plants). However, as summarized above, systemic studies on detailed gene expression profiles in relation to fruit ripening are still very limited. The current information appears to suggest that most of the MAPs kinases are highly expressed in the early development stage, whereas fewer genes exhibit increased expression during fruit ripening. It is likely that the genes with increased expression during fruit ripening may be more tightly connected to the regulation of fruit ripening. Accordingly, loss-of-function and gain-of-function studies on these genes may be able to identify novel MAPs kinase members implicated in the regulation of fruit ripening and quality formation.

### 5.2. Roles of FFP Homologs in the Regulation of Fruit Ripening and Quality Formation

**Figure 2:**
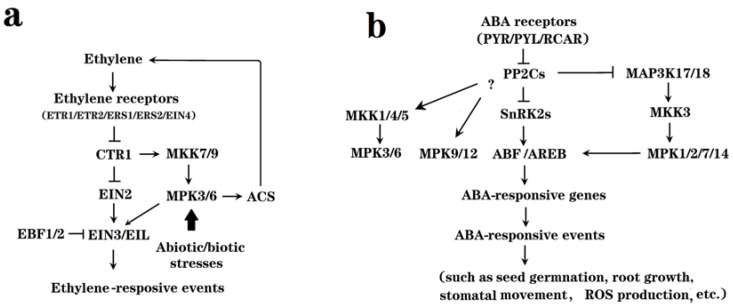
Diagram showing the updated pattern of cellular signal transduction of ET and ABA with emphasis on the involvement of MAPs kinase in *Arabidopsis thaliana*. (**a**) Signal transduction of ET. ET perception by its receptors acts to inhibit CTR1, a Raf-like MAP3K. There exist two signaling pathways downstream of CTR1, i.e., the EIN2 pathway, which acts to control the nuclear accumulation of the EIN3/EIL protein, the core TFs controlling the expression of ET-responsive genes; another is the MAPK signaling pathway, where the MKK7/9-MPK3/6 module acts to link CTR1 with EIN3/EIL. In addition, MPK3/6 act to stabilize ACS via phosphorylation, thereby regulating ET biosynthesis. Noteworthily, MPK3/6 can be also activated by a variety of biotic/abiotic signals. Consequently, the MAPK signaling pathway functions in both mediating ET signal transduction and biotic/abiotic signals modulate ET production. (**b**) Signal transduction of ABA. ABA perception by its receptors acts to inhibit PP2C, clade-A type-2C protein phosphatases. Besides directly targeting SnRK2s, PP2C may also directly target several MAPs kinases, such as MAP3K17/18. In addition, a large number of MAPs kinases, such as MKK1/3/4/5 and MPK1/2/3/6/7/9/12/14, were reported to be involved in ABA signaling, ultimately regulating a variety of biological processes, such as stomatal movement, seed germination, root growth and abiotic/biotic responses.

ET and ABA have been well established to be the two hormones that control CL and NC fruit ripening, respectively. Logically, if different FFP species share similar mechanisms for ET and ABA signal transduction to those in *Arabidopsis*, the FFP homologs of the *Arabidopsis* MAPs kinase should play important roles in fruit ripening. With this in mind, we have summarized the common picture of the involvement of MAPs kinases in ET and ABA signal transduction. As shown in Figure 2, it has been established that many MAPs kinases, such as MKK7/9-MPK3/6, MAP3K17/18, MKK1/3/4/5, MPK1/2/3/6/7/9/12/14, etc., act to mediate ET and ABA signal transduction in *Arabidopsis*. Studies on the FFP homologs of these genes are of particular interest and importance for revealing the molecular mechanisms of fruit ripening and quality formation.

### 5.3. Roles of MAPK Signaling in the Environmental Modulation of Fruit Ripening and Quality Formation

Fruit ripening and quality formation can be greatly modulated by environmental factors; unfortunately, less information is available about the related mechanisms. It has been well established that MAPs kinases play crucial roles in environmental signaling. Given the fact that many MAPs kinases are highly expressed in fruits, changes in the activity of the MAPs kinase resulting from abiotic/biotic stimuli should logically act to affect the signaling cascades, thereby modulating fruit ripening and quality formation. As described above, in comparison with that of *MAPKKK*s, the membership composition of *MAPK*s and *MAPKK*s is much smaller (fewer than 20, commonly around 10). This makes it possible to comprehensively screen for the members that are highly expressed in fruits and sensitively respond to abiotic/biotic stimuli, and this may be able to ultimately identify some novel members of MAPs kinases implicated in the environmental modulation of fruit ripening and quality formation.

### 5.4. Fruit Ripening in Relation to the Regulation of Early Fruit Development

To identify the genes implicated in fruit ripening, a commonly used strategy is to identify the genes that show increased expression profiles during fruit ripening, as mentioned above, whereas the genes implicated in early fruit development have been largely overlooked. In fact, the genes implicated in early fruit development may play more important roles in the determination of fruit ripening. This is because fruit ripening is actually strongly correlated with early fruit development. In strawberries, for example, the seeds (i.e., achenes) act to inhibit fruit ripening owing to the IAA transport from the achenes to the receptacle (i.e., fruit). Because seed formation originates from fruit sets and early fruit development, the genes implicated in seed formation should logically play pivotal roles in the regulation of fruit ripening. As summarized above, regardless of plant species, many MAPs kinase genes show a clearly declining pattern in early fruit development. It is of interest and importance to demonstrate whether the genes with dramatically decreased expression profiles play an important role in the regulation of fruit ripening.

### 5.5. Development of Some Key Technologies Used in Studies on MAPK Signaling in Fruits

Research on cellular signal transduction involves a diverse array of technologies. Besides the technologies commonly used in molecular studies, some specific technologies are required, such as the analysis of protein–protein interaction, kinase activity, identification of phosphorylation sites, protein mutation, protein/DNA interaction, effector/reporter systems, etc. The difficulty of researching signal transduction in fruit cells has been largely ascribed to the lack of related technologies. To conduct research on MAPK signaling in relation to fruit ripening and quality, the development of certain technologies may be required, such as effector/reporter analysis based on transient expression technology for fruit. As stable transgenic systems have not been developed in most horticultural plants, exploitation of effector/reporter analysis based on fruit transient expression should greatly promote the efficiency of identifying the MAPs kinase implicated in fruit ripening and quality formation. Another useful method is multiple gene manipulation, i.e., knocking several genes down/out simultaneously. This is because it is quite common that many MAPs kinases function redundantly, and consequently, the manipulation of a single gene is commonly not able to produce perceptible phenotypes. Although multiple gene manipulation is difficult in most FFP species, it can be realized via CRISPR/Cas9 in tomatoes and strawberries, two model FFPs, respectively, for CL and NC fruits.

## Data Availability

Not applicable.
